# Prognostic value of novel imaging parameters derived from standard cardiovascular magnetic resonance in high risk patients with systemic light chain amyloidosis

**DOI:** 10.1186/s12968-019-0564-1

**Published:** 2019-08-22

**Authors:** Nisha Arenja, Florian Andre, Johannes H. Riffel, Fabian aus dem Siepen, Ute Hegenbart, Stefan Schönland, Arnt V. Kristen, Hugo A. Katus, Sebastian J. Buss

**Affiliations:** 1 Department of Cardiology, Angiology and Pneumology, University of Heidelberg, Im Neuenheimer Feld 410, 69120 Heidelberg, Germany; 2 Department of Cardiology, Kantonsspital Olten, Solothurner Spitäler AG, Baslerstrasse 150, 4600 Olten, Switzerland; 3 Department of Hematology, Oncology and Rheumatology, University of Heidelberg, Im Neuenheimer Feld 410, 69120 Heidelberg, Germany; 4 DZHK (German Centre for Cardiovascular Research), Partner Site Heidelberg/Mannheim, 69120 Heidelberg, Germany; 5 Das Radiologische Zentrum, Radiology Center Sinsheim-Eberbach-Erbach-Walldorf-Heidelberg, Alte Waibstadter Str. 2a, 74889 Sinsheim, Germany

**Keywords:** Immunoglobulin light chain amyloidosis, Cardiovascular magnetic resonance, Long axis strain, Myocardial contraction fraction, Longitudinal function, Prognosis

## Abstract

**Background:**

The differentiated assessment of functional parameters besides morphological changes is essential for the evaluation of prognosis in systemic immunoglobulin light chain (AL) amyloidosis.

**Methods:**

Seventy-four subjects with AL amyloidosis and presence of late gadolinium enhancement (LGE) pattern typical for cardiac amyloidosis were analyzed. Long axis strain (LAS) and myocardial contraction fraction (MCF), as well as morphological and functional markers, were measured. The primary endpoint was death, while death and heart transplantation served as a composite secondary endpoint.

**Results:**

After a median follow-up of 41 months, 29 out of 74 patients died and 10 received a heart transplant. Left ventricular (LV) functional parameters were reduced in patients, who met the composite endpoint (LV ejection fraction 51% vs. 61%, LAS − 6.9% vs − 10%, GLS − 12% vs − 15% and MCF 42% vs. 69%; *p* <  0.001 for all). In unadjusted univariate analysis, LAS (HR = 1.05, *p* <  0.001) and MCF (HR = 0.96, *p* <  0.001) were associated with reduced transplant-free survival. Kaplan-Meier analyses showed a significantly lower event-free survival in patients with reduced MCF. MCF and LAS performed best to identify high risk patients for secondary endpoint (Log-rank test *p* <  0.001) in a combined model. Using sequential Cox regression analysis, the addition of LAS and MCF to LV ejection fraction led to a significant increase in the predictive power of the model (χ^2^ (df = 1) = 28.2, *p* <  0.001).

**Conclusions:**

LAS and MCF as routinely available and robust CMR-derived parameters predict outcome in LGE positive AL amyloidosis. Patients with impaired LV function in combination with reduced LAS and MCF are at the highest risk for death and heart transplantation.

**Electronic supplementary material:**

The online version of this article (10.1186/s12968-019-0564-1) contains supplementary material, which is available to authorized users.

## Introduction

Immunoglobulin light chain (AL) amyloidosis is the most common type of systemic amyloidois. The incidence in developed countries is described about 9–12 cases/million inhabitant per year and autopsy studies suggest that the incidence might even be higher [1–3]. AL amyloidosis is characterized by the extracellular deposition of monoclonal light chains as insoluble and aggregated amyloid fibrils in various tissues, leading to progressive organ dysfunction and death [4, 5]. Commonly affected organs include the heart, kidneys, gastrointestinal tract, liver and the nervous system. Cardiac involvement is described in up to 50% of AL amyloidosis patients during the course of the disease [6], and is the most important determinant of clinical outcome [7]. The median survival in patients with heart failure symptoms is about 6 months [8–10]. For prediction of outcome, especially the two biomarkers, N-terminale natriuretic peptide (NT-proBNP) and cardiac Troponin-T (cTNT) are established [11–13]. Based on these biomarkers the widely used Mayo Clinic staging was developed for risk stratification of AL patients [11, 14].

**Fig. 1:**
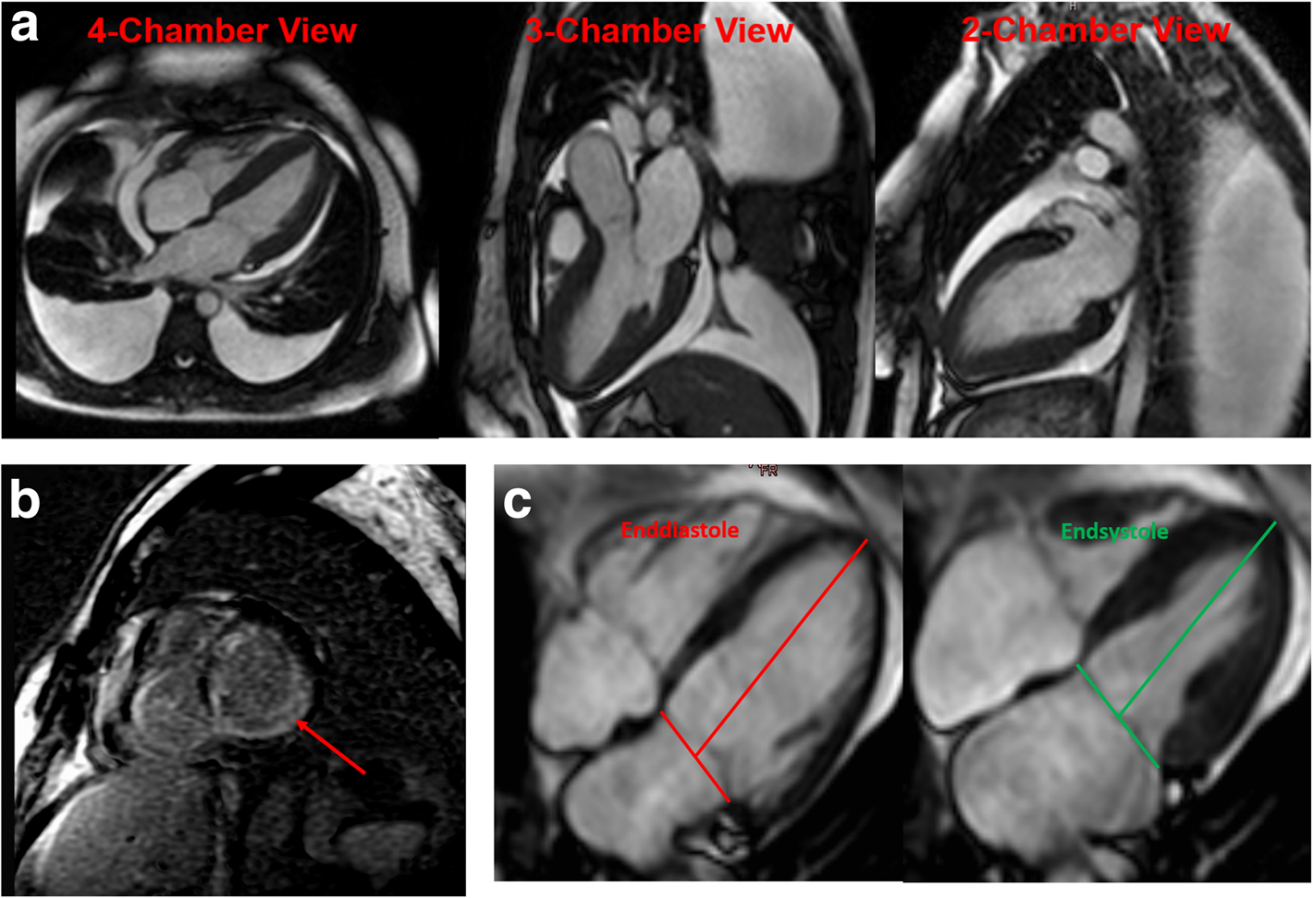
Representative cardiovascular magnetic resonance (CMR) images of **a**) a patient with light chain (AL) amyloidosis demonstrating global left ventricular (LV) wall hypertrophy, pericardial effusion and both-sided pleural effusions, **b** Late gadolinium enhancement (LGE) pronounced in the subendocardial layers in cardiac amyloidosis (marked with a red line) and **c**) long axis strain (LAS) measurement

Beside development of heart failure symptoms, amyloidosis may initially lead to apparent left ventricular (LV) wall thickening mimicking left ventricular hypertrophy (LVH). Cardiovascular magnetic resonance (CMR) imaging is recommended as the diagnostic tool of choice in LVH as it can help differentiate cardiac amyloidosis from other potential causes of a cardiomyopathy (Fig. 1a) [15–17]. The presence of global diffuse myocardial late gadolinium enhancement (LGE) pronounced in the subendocardial layers is common in cardiac amyloidosis (Fig. 1b), and has been associated with poor prognosis [18]. Besides LGE, only few other markers, such as global longitudinal strain (GLS), are available for risk stratification in AL patients [19].

Therefore, this study aims to assess the novel CMR-derived deriveparameters long axis strain (LAS) and myocardial contraction fraction (MCF) for risk stratification in patients with AL amyloidosis. Both parameters have several important advantages. First, they can be derived from every standard CMR examination without the need for a dedicated post processing software. Second, the assessment of LAS and MCF does not require gadolinium contrast, which is relatively contraindicated in patients with severe impaired renal function. Third, unlike biomarkers, LAS and MCF are not influenced by any organ dysfunction, which may exist in systemic amyloidosis.

## Methods

### Study population and design

The goal of this study was to evaluate the prognostic value of novel imaging parameter in biopsy-proven and LGE-positive AL amyloidosis patients.

The study population consisted of consecutive patients with AL amyloidosis, who received a CMR scan between June 2005 and October 2014 at the University of Heidelberg as part of a standard institutional protocol for the evaluation of cardiomyopathies. CMR was performed in all patients with AL amyloidosis in a clinically stable condition.

Only patients with biopsy-proven AL amyloidosis and LGE pattern characteristic for cardiac amyloidosis were included in the analysis. Exclusion criteria were contraindications for CMR including incompatible devices such as cardiac pacemakers or implantable cardioverter defibrillators (ICD) or other metallic implants, severe claustrophobia, severe obesity preventing patient entrance into the scanner bore, and pregnancy or lactation. Patients with renal failure and an estimated glomerular filtration rate (eGFR) < 30 ml/min/1.73m^2^ were also excluded from the analysis.

This single center study was approved by the local Ethics Committee and in accordance with the Declaration of Helsinki. The study population signed a written informed consent for the retrospective post-hoc analysis of their clinical routine data.

### Adjudication of the diagnosis

In all patients, the diagnosis of systemic AL amyloidosis was confirmed by biopsy and the cardiac involvement showed a characteristically LGE pattern and LV morphology.

Diagnosis of AL amyloidosis was based on tissue deposition of amyloid, the presence of a monoclonal gammopathy by serum electrophoresis, immunofixation on serum and urine, with free light chains, and conformation by positive Congo red staining with birefringence under polarized light of any biopsy (periumbilical fat aspiration, rectum, or target organ), positive immunohistology for kappa or lambda in the biopsy, and on the exclusion of hereditary forms of amyloidosis, if necessary.

### Blood samples

Renal function, NT-proBNP, cTNT and the difference between pathologic and nonpathologic serologic free light chains (dFLC) were determined [20]. Because of laboratory changes of the clinical routine from conventional cTNT to high sensitivity cTNT (hs cTnT), a part of study population has conventional cTNT (*n* = 34) the other hs cTNT (*n* = 40). The samples for NT-proBNP and cTNT were analyzed using the commercially available sandwich immunoassay on a fully automated analyzer (ELECSYS, Roche Diagnostics, Mannheim, Germany). eGFR was calculated in each individual using the “Modified Diet in Renal Diseases” (MDRD) formula [21].

### CMR protocol

All CMR scans were performed on a 1.5 T CMR system (Achieva™, Philips Healthcare Best, The Netherlands) using an institutional standard protocol. A 32-channel phased-array cardiac coil was used. Cine images were obtained using a breath-hold, segmented-k-space, balanced steady-state free precession sequence with retrospective electrocardiogram (ECG) gating in long axis views (2, 4 and 3 chamber) as well as in contiguous short axis slices covering the entire LV and right ventricle (RV) from the annulus of the atrioventricular valves to the apex, with 35 phases per cardiac cycle. The CMR imaging parameters were: field-of-view (FOV) = 350 × 350 mm^2^, repetition time/echo time (TR/TE) = 2.8/1.4 ms, acquired voxel size = 2.2 × 2.2 × 8 mm^3^, flip angle (FA) = 60°, reconstructed voxel size = 1.3 × 1.2 × 8 mm^3^. Data were analyzed by a single examiner blinded to the patient’s clinical status. The analysis was performed on a commercially available clinical workstation (IntelliSpace Portal (ISP) Version 7.0.1, Philips Healthcare). Results for LV volumes, LV ejection fraction (LVEF) and LV myocardial mass were derived from short axis slices by tracing endocardial and epicardial borders of the LV. Papillary muscles and trabeculations were excluded from LV mass. LGE imaging acquisitions were performed 10 min after the administration of 0.2 mmol Gd-DTPA/kg body weight (Magnevist, Schering, Germany). For selection of appropriate inversion time (TI) gradient echo sequences were used to null myocardial signal. The TI scout had the following parameters: TE 2.0 milliseconds, TR 3.4 milliseconds and flip angle 25°. Three-dimensional LGE sequences were carried out during breath-hold in expiration and using retrospective ECG gating. The pattern of LGE was described as transmural if LGE involved circumferential the complete subendocardium through to the epicardium. The presentation of subendocardial involvement was specified as subendocardial LGE. The term patchy and focal LGE was used for description of any other LGE distribution, for example a mid-wall, subepicardial or diffuse.

### Assessment of long Axis strain (LAS) and myocardial contraction fraction (MCF)

LAS is an image based functional marker that describes longitudinal function. LAS is defined as the percentage in longitudinal shortening of the LV between end-diastole and end-systole (Fig. 1c). To calculate LAS we utilized the strain formula:

$$ LAS=\frac{l_{sys}-{l}_{dias}}{l_{dias}}\bullet 100\ \left[\%\right] $$

MCF was calculated by dividing LV stroke volume (LV end-diastolic volume - LV end-systolic volume) by LV myocardial volume:

$$ MCF=\frac{LV\  Stroke\ volume\ (ml)}{LV\  Myocardial\ Volume\ (ml)}\ast 100\ \left[\%\right] $$

LV myocardial volume was defined as LV myocardial mass divided by the mean density of myocardium, which is 1.05 g/ml. To achieve the index the result was multiplied by 100.

Both parameters have been validated for their diagnostic and prognostic value in previous studies [22–25].

### Assessment of longitudinal strain using feature tracking imaging (FTI)

CMR myocardial global strain analyses were performed using dedicated 2D CPA CMR Feature tracking software (TomTec Imaging Systems, Munich, Germany). This software algorithm has been validated previously in experimental and clinical studies [26–28]. The LV endocardial borders of a 4-chamber view were used for the calculation of the longitudinal strain and an automatic tracking was performed throughout the complete cardiac cycle. If the automatic tracking was incorrect, the contours were manually adjusted. Measurements were repeated three times and then averaged, resulting in the mean segmental strains and GLS. The technique has been described in detail recently [29].

### Outcomes and follow-up

All-cause mortality was the primary study endpoint. The combination of all-cause mortality and heart transplantation (HTX) due to progressive disease was defined as the secondary endpoint. Follow-up was obtained by review of the patient’s hospital chart or telephone interview with the patient or relatives. All survivors completed the 2 years of follow-up. The median follow up of surviving patients was 55 months (43–66 months).

### Statistics

Categorical variables are given as number and percentage, continuous parametric variables as mean ± standard deviation (SD) and continuous non-parametric variables as median and interquartile range (IQR). For the comparison of means between groups two-tailed Student’s t-test was used and differences between nominal variables were assessed using the Fisher exact test. Group differences of continuous non-parametric variables were tested using the nonparametric Mann-Whitney U test. Proportions of categorical were compared using Chi-squared test. Correlations were analysed using Spearman’s rank correlation coefficient. Kaplan-Meier curves were used to estimate the distribution of survival as a function of the follow-up duration. Optimal cut-off values were defined by Receiver operating characteristics (ROC) and Youden’s J statistic. The association of clinical, imaging and serological parameters with outcome was evaluated by uni- and multivariate Cox proportional-hazards regression models. Differences were considered statistically significant at *p* <  0.05. All statistics were calculated using MedCalc 15 (MedCalc™, Mariakerke, Belgium).

## Results

### Baseline characteristics

Initially 94 patients with systemic AL amyloidosis were included in the study. However, 20 patients, who received chemotherapy before CMR was performed, were excluded from the final analysis. Therefore, the total study consists of 74 subjects.

Chemotherapy was administered 65 days (22–106 days) after CMR examination. 15 participants (20.3%) received melphalan and dexamethasone, 16 (21.6%) bortezomib and further 43 (58.1%) received other forms and combinations of chemotherapy. The amount of organ involvement was heterogenic (patient number: organs involved: 16:1, 25:2, 20:3, 6:4 and 7:5).

During the follow-up period of 2 years, 29 patients died, while 10 patients (13.5%) received a HTX. After the follow-up period, 35 patients were alive without HTX (47.3%), leaving 39 patients (52.7%), who reached the combined endpoint of death or HTX. Baseline characteristics including co-morbidities, laboratory data and CMR measurements are presented in Table 1.

**Table 1:** Demographics and cardiovascular magnetic resonance (CMR) data of patients with light-chain amyloidosis (*n* = 74), with and without reaching the composite endpoints during the follow-up of 2 years

CMR Data	All (*n* = 74)	Transplant-free survivors (*n* = 35)	Composite endpoint (death or heart transplantation) (*n* = 39)	*p*-Value (between transplant free survivors and composite endpoint)
Age (years)	58.5 ± 10.8	58.2 ± 9.8	58.7 ± 11.7	0.84
BMI (kg/m^2^)	25.9 ± 4.5	26.4 ± 4.8	25.5 ± 4.2	0.4
Male gender, n (%)	50 (67.6)	21 (60)	29 (74.4)	0.2
Cardiovascular risk factors, n (%)				
Arterial hypertension	26 (35)	11 (31.4)	16 (41)	0.31
Dyslipidemia	10 (13.5)	6 (17.1)	4 (44)	0.45
Diabetes mellitus	7 (9.5)	5 (14.3)	2 (5.1)	0.27
Smoking	12 (16.2)	5 (14.3)	7 (17.9)	0.67
Family history of sudden cardiac death	4 (5.4)	1 (2.9)	3 (7.7)	0.4
Clinical data				
NYHA class, n (%)				0.03
I	15 (20.3)	11 (31.4)	4 (10.2)	
II	23 (31.1)	12 (34.3)	11 (28.2)	
III	36 (48.6)	12 (34.3)	24 (61.5)	
Karnofsky index	79.5 ± 11.5	84.6 ± 7.6	74.9 ± 12.5	< 0.001
Laboratory data				
Lambda restricted pts.	60 (81.1)	26 (74.3)	34 (81.2)	0.3
Kappa restricted pts.	14 (18.9)	9 (25.7)	5 (12.8)	0.29
dFLC (mg/dL)	222.3 ± 110	108.9 ± 77.9	321.2 ± 197.3	< 0.001
MDRD (mL/min/1.73m^2^)	68.3 ± 24.7	71.1 ± 23.7	65.7 ± 25.9	0.36
Positive Troponin, n (%)	53 (71.6)	19 (54.3)	34 (87.2)	0.002
ln NT-pro BNP	7.6 ± 1.8	6.4 ± 1.9	8.4 ± 1.2	< 0.001
CMR Data				
LAS (%)	−8.4 ± 3.7	−10 ± 3.7	−6.9 ± 3.2	< 0.001
MCF (%)	54.9 ± 26.2	69.3 ± 27.5	42 ± 15.7	< 0.001
LVEDVI (ml/m^2^)	72.2 ± 18.7	72.6 ± 19.1	71.8 ± 18.6	0.89
LVESVI (ml/m^2^)	32.5 ± 14	28.8 ± 11.6	35.9 ± 15.2	0.03
LVEF (%)	55.6 ± 11.9	60.5 ± 11.5	51.2 ± 10.7	< 0.001
LVMI (g/m^2^)	84.8 ± 29	71.5 ± 20.2	96.8 ± 30.7	0.014
GLS (%)	−13.7 ± 5.5	−15.4 ± 5.9	− 12.2 ± 4.7	0.011
Basal longitudinal strain (%)	−12.8 ± 10.7	−14.3 ± 11.9	−11.5 ± 9.5	0.27
Midwall longitudinal strain (%)	−17.2 ± 11.7	−18.3 ± 12.7	−16.2 ± 10.8	0.46
Apical longitudinal strain (%)	−22.4 ± 11.1	−23.1 ± 12	−21.8 ± 10.2	0.63

*Abbreviations*: *dFLC* free light chain difference, *GLS* global longitudinal strain, *LAS* long axis strain, *LVEDVI* left ventricular end-diastolic volume index, *LVESVI* left ventricular end-systolic volume index, *LVEF* left ventricular ejection fraction, *LVMI* left ventricular mass index, *MCF* myocardial contraction fraction, *NYHA* New-York-Heart-Association, *NT – pro BNP* N-terminale natriuretic peptide

The age of all study participants was 59 ± 11 years and 50 (68%) subjects were male. Cardiovascular disease risk factors were presented in both groups (alive vs. death/HTX) without any significant difference (Table 1). Arterial hypertension was the most common risk factor (35%). Nearly half of the patients presented with dyspnea New-York-Heart-Association (NYHA) class III (49%). The number of patients with lambda light chains (*n* = 60, 81.1%) was higher than the one of kappa light chains (*n* = 14, 18.9%). The values of dFLC (108.9 ± 78 vs. 321.2 ± 197.3, *p* <  0.001) and NT-pro BNP were higher in patients, who reached the combined endpoint. In addition, a high proportion of the total study population presented positive values of cTNT (71.6%).

CMR measurements of LV end-systolic volume, LVEF, mass as well as strain analysis demonstrated a significant difference in patients who met the composite endpoint (Table 1). Standard two-dimensional (2D) GLS was reduced in all AL patients (− 13.7 ± 5.5%) with a significant difference in both groups of the secondary composite endpoint (− 15.4 ± 5.9% vs. -12.2 ± 4.7%, *p* = 0.011). In addition, the segmental longitudinal strain analysis demonstrated normal longitudinal strain of the apical segments, while basal segment strain segments were significantly reduced (longitudinal strain values of basal segments: − 12.8 ± 10.7, midwall segments: − 17.2 ± 11.7 and apical segments: − 22.4 ± 11.1, *p* <  0.01). However, the segmental analysis did not significantly differ between both groups (Table 1).

### Correlation analysis

There was an intermediate correlation between MCF and LAS (Spearman’s coefficient of rank correlation/rho: − 0.72, *p* <  0.0001). In addition, the correlations between the CMR-derived parameters (MCF and LAS) and cardiac biomarkers (NT-proBNP and cTNT) was weak to moderate (Spearman’s coefficient of rank correlation/rho between: 1. MCF and NT-proBNP − 0.6, *p* <  0.0001; 2. MCF and cTNT − 0.41, *p* <  0.001; 3. LAS and NT-proBNP 0.59, *p* <  0.001; 4. LAS and cTNT 0.27, *p* = 0.006).

### Survival analysis

In the unadjusted univariate analysis NYHA class, the Karnofsky index, cardiac biomarkers and parameters of myocardial morphology and function as assessed by CMR were significantly associated with overall survival (Table 1). Especially, measurements of the LV function, including LAS (Hazard ratio (HR) = 1.2, *p* <  0.001), GLS (HR = 0.92, *p* = 0.04), LV mass index (LVMI) (HR = 1.02, *p* <  0.001), LV ejection fraction (LVEF) (HR = 0.97, *p* = 0.02) and MCF (HR = 0.96, *p* <  0.001) were all associated with a reduced survival in patients with AL amyloidosis (Table 2 and 3).

**Table 2:** Univariate analysis of all patients (*n* = 74) for primary endpoint (death)

		Primary endpoint	
Variable	Hazard ratio	95% CI	*p*-value
NYHA class	1.8	1.1–2.9	0.02
Karnofsky Index	0.94	0.92–0.97	< 0.001
dFLC (mg/dL)	1.0	0.99–1.01	0.27
Positive Troponin values	2.3	1.2–4.7	0.02
ln NT-pro BNP	2.8	0.98–8.1	0.06
LAS (%)	1.2	1.1–1.4	< 0.001
MCF (%)	0.96	0.94–0.98	< 0.001
LVESVI (ml/m^2^)	1.01	0.99–1.04	0.13
LVEF (%)	0.97	0.94–0.99	0.02
LVMI (g/m^2^)	1.02	1.01–1.03	< 0.001
GLS (%)	0.92	0.85–0.99	0.04

*Abbreviations*: *dFLC* free light chain difference, *GLS* global longitudinal strain, *LAS* long axis strain, *LVESVI* left ventricular end-systolic volume index, *LVEF* left ventricular ejection fraction, *LVMI* left ventricular mass index, *MCF* myocardial contraction fraction, *NYHA* New-York-Heart-Association, *NT-pro BNP* N-terminale natriuretic peptide

**Table 3:** Univariate analysis of all patients (*n* = 74) for composite endpoint (death and heart transplantation)

		Composite endpoint	
Variable	Hazard ratio	95% CI	*p*-value
NYHA class.	1.9	1.2–3.0	0.006
Karnofsky Index	0.95	0.93–0.98	< 0.001
dFLC (mg/dL)	1.0	0.99–1.01	0.2
Positive Troponin values	2.5	1.3–4.6	0.005
ln NT-pro BNP	3.8	1.3–11	0.01
LAS (%)	1.23	1.1–1.4	< 0.001
MCF (%)	0.96	0.94–0.99	< 0.001
LVESVI (ml/m^2^)	1.04	1.02–1.04	0.006
LVEF (%)	0.95	0.92–0.97	< 0.001
LVMI (g/m^2^)	1.01	1.0–1.03	< 0.001
GLS (%)	0.91	0.85–0.97	0.005

*Abbreviations*: *dFLC* free light chain difference, *GLS* global longitudinal strain, *LAS* long axis strain, *LVEDVI* left ventricular end-diastolic volume index, *LVESVI* left ventricular end-systolic volume index, *LVEF* left ventricular ejection fraction, *LV MI* left ventricular mass index, *MCF* myocardial contraction fraction, *NYHA* New-York-Heart-Association, *NT – pro BNP* N-terminale natriuretic peptide

In a stepwise multivariate Cox regression model only MCF and Karnofsky Index were independent predictors for the primary endpoint (Table 4). While, MCF, LAS and NT-proBNP remained independent predictors for the composite endpoint (Table 5). Therefore, MCF alone was associated with a reduced survival in both, primary and composite, endpoints.

**Table 4:** Multivariate proportional-hazard model for primary endpoint (death)

		Endpoint	
Variable	Hazard ratio	95% CI	*p*-value
MCF	0.97	0.96–0.99	0.002
Karnofsky Index	0.96	0.93–0.99	0.006

*Abbreviations*: *MCF* myocardial contraction fraction

**Table 5:** Multivariate proportional-hazard model for composite endpoint (death and heart transplantation)

		Endpoint	
Variable	Hazard ratio	95% CI	*p*-value
MCF	0.96	3.6–32.4	< 0.001
LAS	1.05	0.94–0.98	< 0.001
ln NT-pro BNP	5.2	1.8–15,3	0.003

*Abbreviations*: *LAS* long axis strain, *MCF* myocardial contraction fraction, *NT – pro BNP* N-terminale natriuretic peptide

**Fig. 2:**
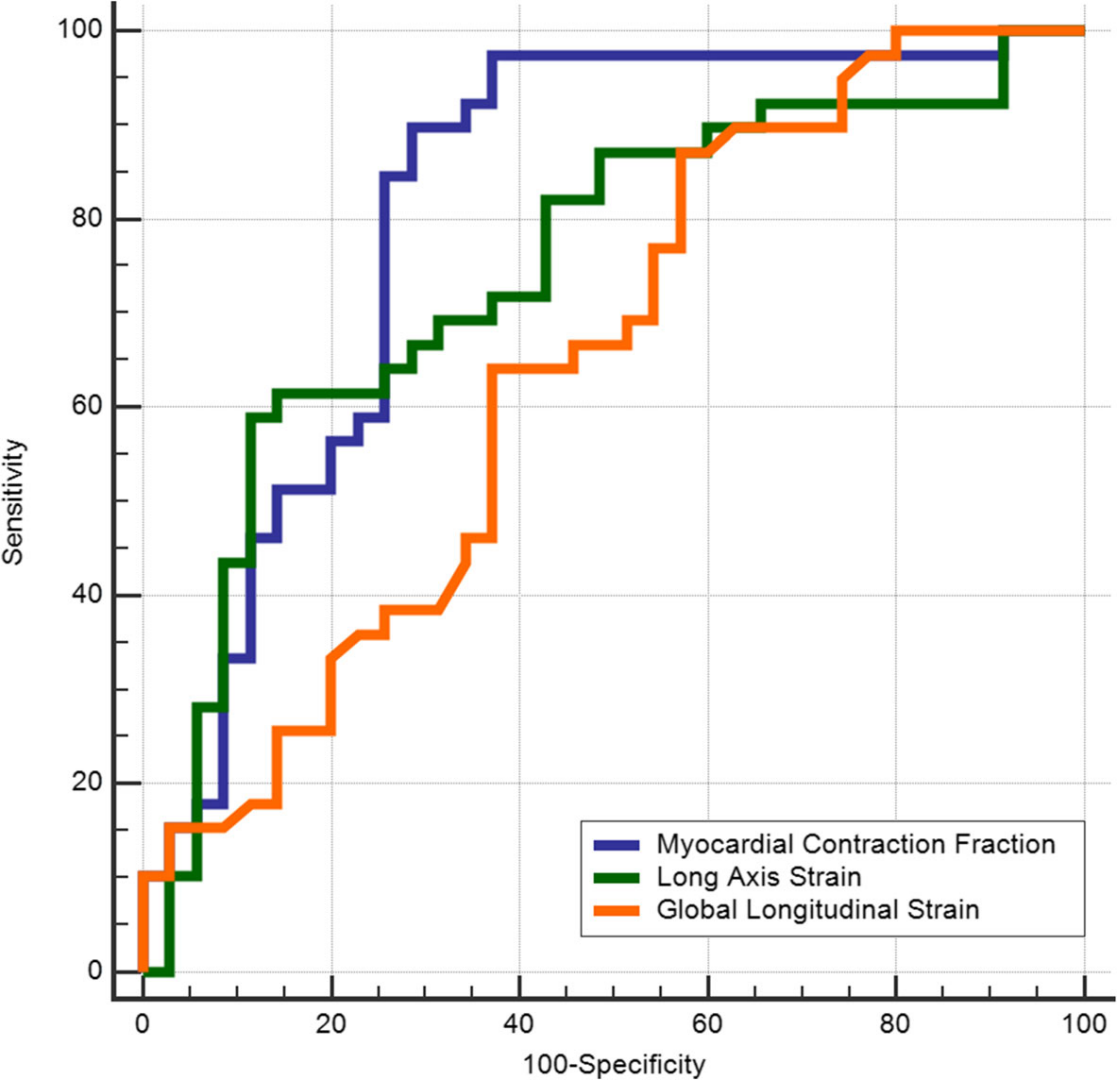
Receiver operating characteristic (ROC) curves for myocardial contraction fraction (MCF), long axis strain (LAS) and global longitudinal strain (GLS) in AL amyloidosis patients for the combined the composite endpoint (death and heart transplantation)

MCF featured the largest area under the curve (AUC = 0.81) based on the ROC curve analysis regarding the secondary endpoint. The AUC for LAS was 0.75 and for GLS was 0.65. A significant difference was found between the fields under the ROC curves for MCF and GLS (*p* = 0.012, Fig. 2).

### Risk stratification in AL amyloidosis

**Fig. 3:**
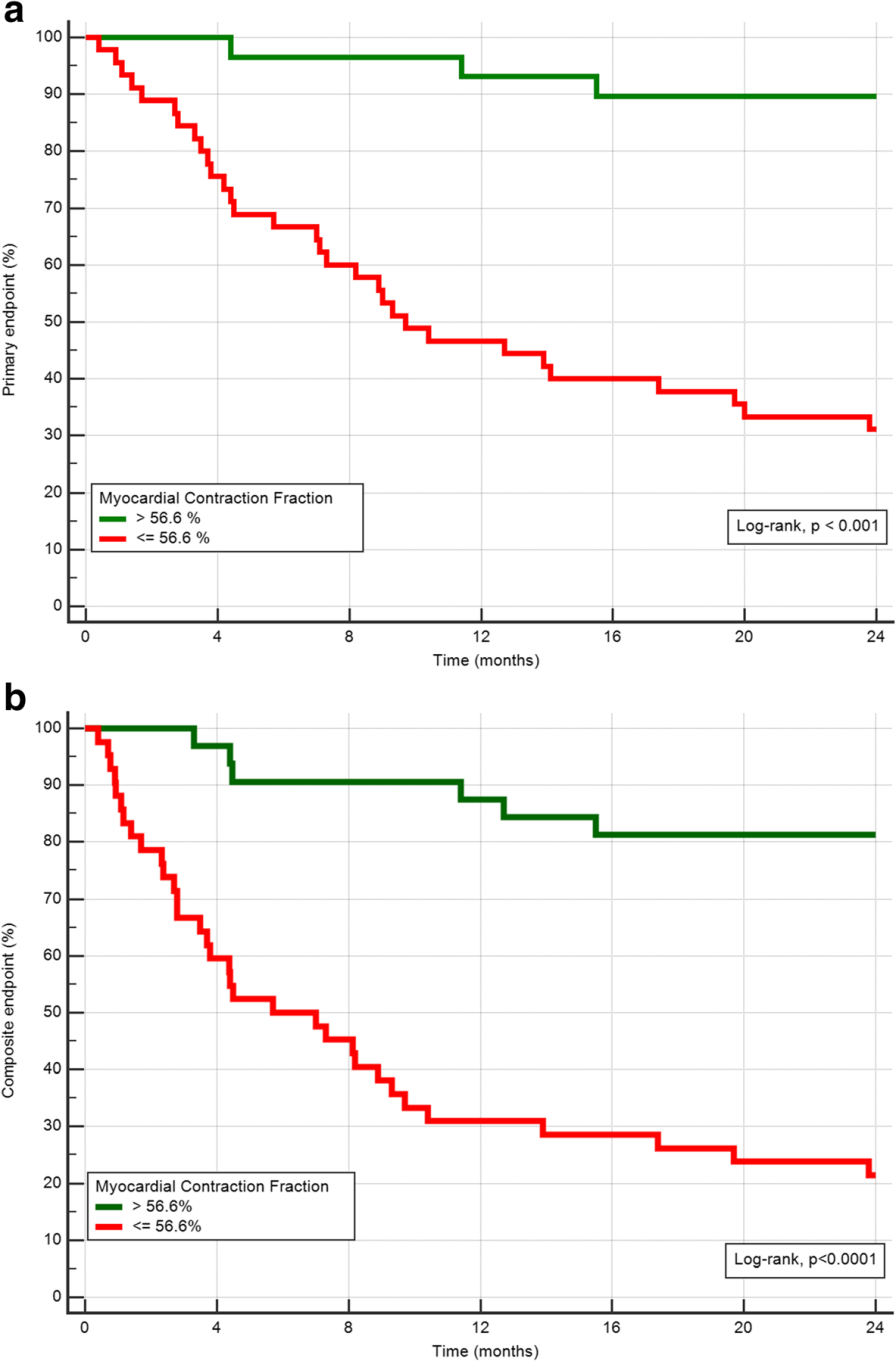
**a**) Kaplan-Meier estimates of the time to events by myocardial contraction fraction (MCF) optimized cut-off 56.6% for primary endpoint (death) and **b**) for the composite endpoint (death and heart transplantation)

**Fig. 4:**
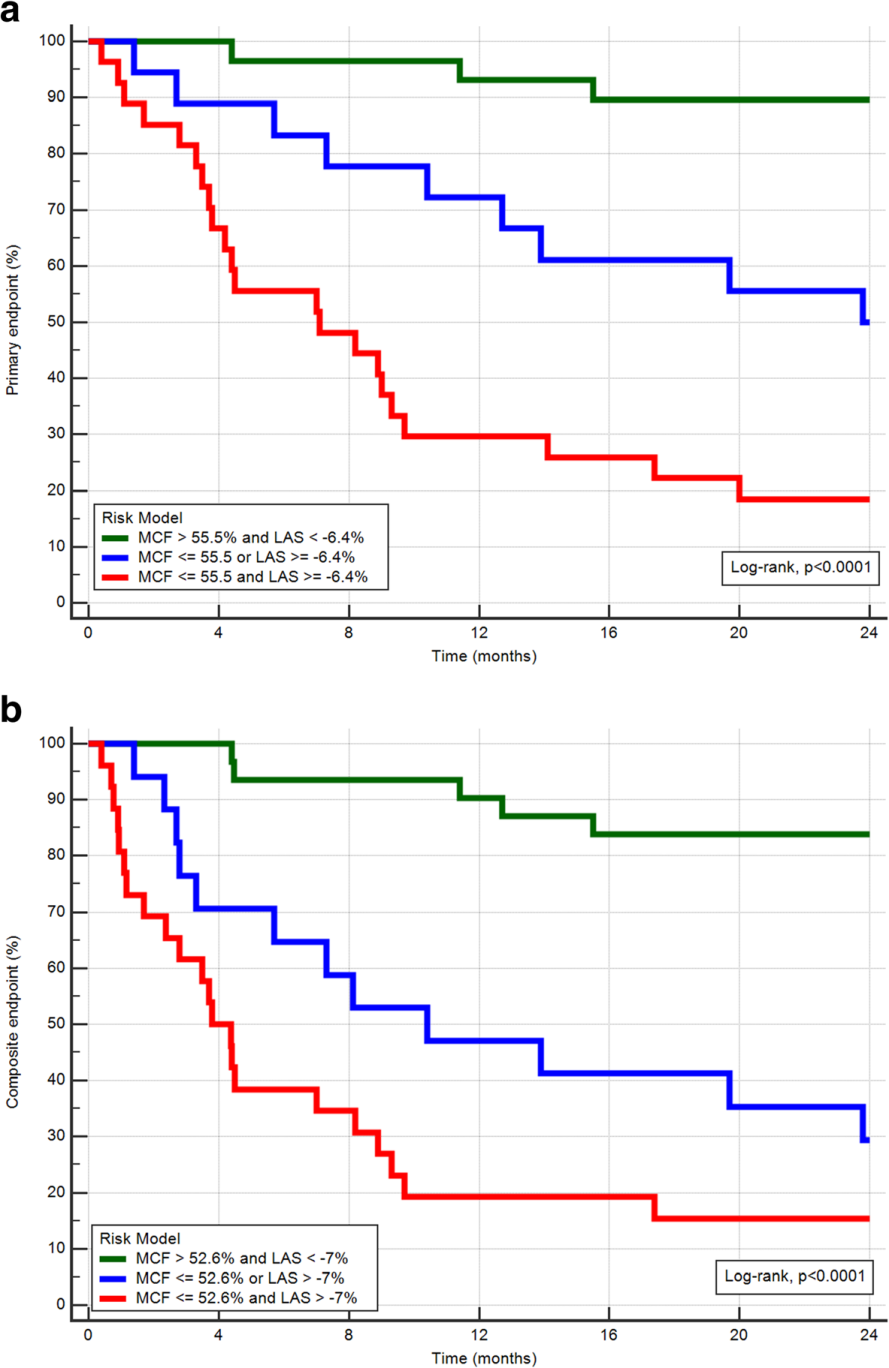
Kaplan-Meier estimates of the time to events by myocardial contraction fraction (MCF) and longitudinal axis strain (LAS). Presented **a**) for primary endpoint (death) and **b**) for the composite endpoint (death and heart transplantation)

The Kaplan-Meier analyses for primary and for composite endpoint demonstrated a significantly reduced event-free survival in patients with a LAS value above − 7% and a MCF value below 56.6% (Log-rank test *p* <  0.001, Fig. 3a and b). The combined predictor model including MCF and LAS, could better risk stratify patients regarding the primary and secondary endpoint (Fig. 4a and b).

**Fig. 5:**
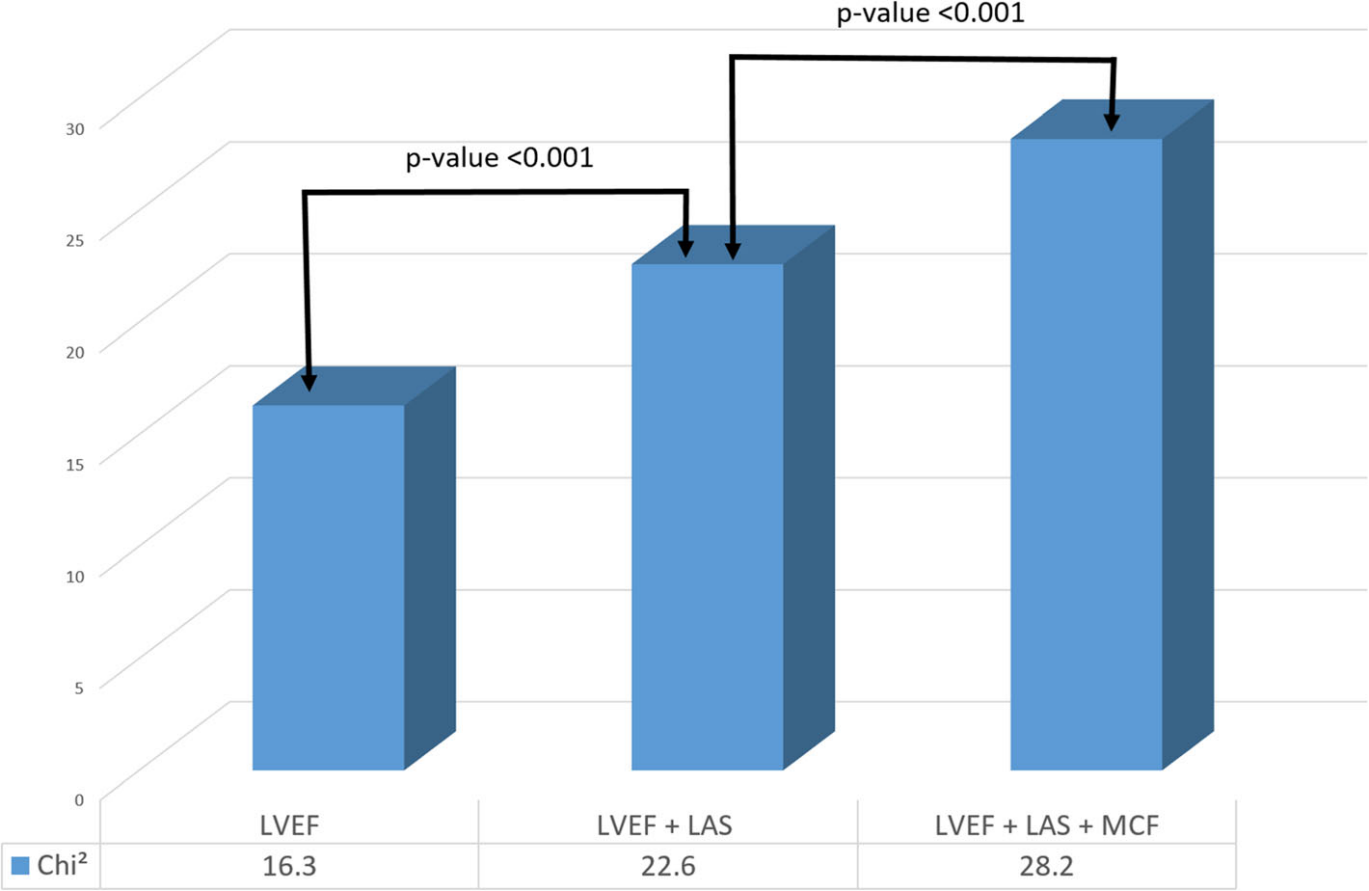
Incremental predictive value of myocardial contraction fraction (MCF) and longitudinal axis strain (LAS) to left ventricular ejection fraction (LVEF) regarding the combined endpoint AL amyloidosis patients

Using a sequential Cox regression analysis for an imaging based predictor model, the addition of LAS to a model including LVEF (χ^2^ (df = 1) = 16.3) led to a significant increase in the predictive power (χ^2^ (df = 1) = 22.6, *p* <  0.001). The addition of MCF to this model resulted in a further significant increase in the predictive power (χ^2^ (df = 1) = 28.2, *p* <  0.001) (Fig. 5).

### Subgroup LGE analysis in AL amyloidosis

The complete study population presented a LGE distribution characteristically for cardiac amyloidosis. However, the analysis of the exact distribution pattern was not possible in the complete study population, which is conditioned by the challenge in choosing the appropriate TI. Therefore, only 70 of 74 (95%) LGE images were available for a reliable analysis. Our analysis demonstrated a trend of higher mortality rates in patients presenting transmural LGE. However, we found no significant difference between the LGE pattern and the endpoint (Additional file 1: Table S6, Additional file 2: Figure S6). Furthermore, there was no significant difference between the mean values of the CMR parameter in the different LGE groups (group 1: subendocardial LGE, *n* = 26 (37.1%), group 2: transmural LGE, *n* = 25 (35.7%) and group 3: patchy and focal LGE, *n* = 19 (27.1 Additional file 1: Table S7).

## Discussion

To our knowledge, this is the first study assessing two novel CMR-derived imaging parameters for risk prediction in patients with systemic AL amyloidosis, who already presented with cardiac involvement and the presence of LGE. The determination of LAS and MCF can further risk stratify these subjects. AL amyloidosis patients with a restricted LV function combined with reduced LAS (> − 7%) and MCF (≤ 52.6%) values were at greatest risk for death.

Cardiac involvement of AL amyloidosis is a major determinant of treatment options and prognosis. Early identification of high-risk patients may lead to begin of intensive therapeutic strategies with better survival. Cardiac serum biomarkers NT-proBNP and cTNT are currently used in clinical routine for estimation of the progno*s*is in patients with cardiac involvement. Previous studies have shown a significant correlation between LGE extent on CMR and biomarkers of myocardial injury in patients with acute viral myocarditis [30].

CMR is currently the diagnostic tool of choice for identification of cardiac amyloidosis. Additionally, it has been shown, that presence of LGE in patients with systemic amyloidosis is associated with mortality [31, 32]. However, myocardial LGE, pronounced in the subendocardial layers, is common in cardiac amyloidosis and thus, further parameters are of great clinical interest. Furthermore, the application of contrast agents is frequently contraindicated, due to reduced renal function. In addition, our subgroup analysis could not show any difference regarding outcome and LGE pattern. Therefore, there is a need for additional parameters, such as LAS and MCF, which can contribute to the identification of very high-risk amyloidosis patients.

Previous studies have already shown that LV longitudinal function may be a marker for early diagnosis and outcome in cardiac diseases of various etiologies [33–35]. Especially, the assessment of LV longitudinal function by strain using tissue Doppler echocardiography has been described to identify early impairments of LV function in AL amyloidosis [36, 37]. In addition, the longitudinal function by strain analysis was associated with poor prognosis and outperformed standard echocardiographic parameters suggesting that strain imaging could serve as a new tool to identify high risk subjects [19, 38, 39]. Our study results are in agreement with previous data. First, longitudinal strain derived from cine CMR imaging demonstrated the specific amyloidosis pattern with normal apical and reduced strain values of the basal segments, and second, impaired GLS was associated with reduced outcome. However, in a stepwise multivariate Cox regression model LAS and MCF outperformed GLS [40].

CMR-derived LAS has been shown to be a simple and rapidly assessable parameter representing global LV longitudinal function without the necessity of additional post-processing software tools [24]. The major advantages of LAS are its independence of dedicated software and its very good intra- and interobserver variability [24]. The diagnostic and prognostic value of LAS was evaluated in non-ischemic cardiomyopathies (NICM) [23, 25]. Previous analysis demonstrated not only a significantly better performance of LAS than LVEF and mitral annular plane systolic excursion (MAPSE) in discriminating controls from NIDCM, but also a significantly higher rate of cardiac events in NICM patients with reduced LAS values, independent of the presence of LGE [25]. Assessment of longitudinal function with LAS offered incremental information for the prediction of cardiac events in NICM and improved risk stratification beyond established CMR parameters such as LVEF and the presence of myocardial fibrosis. These results were further confirmed in a recent publication on LAS in NICM [23]. The current analysis could demonstrate that CMR-derived LAS may also serve as reliable prognostic marker in other cardiomyopathies, who are known of reduced LV longitudinal contractility. In a further study Doesch et al., showed a reduced LV longitudinal shortening in hypertrophic cardiomyopathy (HCM) patients measured by CMR-assessed MAPSE compared to healthy controls [41].

MCF is a quantitative parameter representing a volumetric index of fractional contraction of the myocardium. MCF is calculated as a ratio between LV stroke volume and the LV myocardial volume. In LVH independent of its etiology, a decrease in MCF indicates an abnormal myocardial function, although LVEF may remain normal even in advanced stages because of the progressive reduction in ventricular capacitance. Therefore, measurement of LVEF alone gives an incomplete representation of the complex process of dysfunction especially in hypertrophied hearts. In a previous study we could demonstrate that CMR-derived MCF has an excellent diagnostic accuracy discriminate between patients with AL amyloidosis from patients with other forms of LVH [22]. We could define cut-off value for MCF of < 50%, which allowed to identify patients with a high probability for cardiac amyloidosis. The prognostic value of MCF in patients with cardiac amyloidosis (34 AL and 32 ATTR subjects) was studied by Tendler et al. [42]. The authors reported a superiority of MCF to LVEF in predicting survival of patients with AL amyloidosis. However, in this study MCF was assessed from 2D echocardiography data, which are known for their limited accuracy and reproducibility, especially when compared to CMR as the accepted “gold standard” of LV mass measurements [43]. Even the more recent approaches using 3D echocardiography show only limited performance compared to CMR, suffering from substantial variability and underestimation [16, 44]. Therefore, we assume that MCF derived from CMR images is more accurate and reproducible.

The present study has some limitations. First, it is a retrospective analysis. Second, because of two different Troponin essays (conventional and high-sensitivity) in our institution during the study period, it was only possible to divide between troponin positive and negative participants. Therefore, an assessment regarding Troponin values and outcome was not possible. Finally, because of a long period of data collection beginning from 2005, this study does not provide any T1 mapping information.

## Conclusions

In conclusion, LAS and MCF are easily available and robust CMR-derived parameters which predict outcome in LGE-positive AL amyloidosis patients. Among these, patients with reduced LAS and MCF fraction are at highest risk for death and HTX.

## Additional files

Additional file 1: **Table S6.** Late Gadolinium Enhancement (LGE) pattern analysis. **Table S7.** CMR data of patients with light-chain amyloidosis compared to different Late Gadolinium Enhancement (LGE) distribution groups (DOCX 31 kb)

Additional file 2: **Figure S6.** Kaplan-Meier estimates of the time to events by late gadolinium enhancement (LGE) pattern. Presented for the composite endpoint (death and heart transplantation). (PNG 33 kb)

## Data Availability

The datasets used and analyzed during the current study are available from the corresponding author on reasonable request.

## Abbreviations

AL: Immunoglobulin light chain
AUC: Area under the curve
bSSFP: Balanced steady-state free precession
CMR: Cardiovascular magnetic resonance
cTNT: Cardiac troponin-T
ECG: Electrocardiogram
eGFR: Estimated glomerular filtration rate
FA: Flip angle
FOV: Field-of-view
GCS: Global circumferential strain
GLS: Global longitudinal strain
HCM: Hypertrophic cardiomyopathy
Hs cTNT: High sensitivity cardiac troponin-T
HTX: Heart Transplantation
ICD: Implantable cardioverter defibrillator
IQR: Interquartile range
LAS: Long axis strain
LGE: Late gadolinium enhancement
LV: Left ventricle/left ventricular
LVEDVI: Left ventricular end-diastolic volume index
LVEF: Left ventricular ejection fraction
LVESVI: Left ventricular end-systolic volume index
LVH: Left ventricular hypertrophy
LVMI: Left ventricular mass index
MAPSE: Mitral annular plane systolic excursion
MCF: Myocardial contraction fraction
MDRD: Modified diet in renal diseases
NICM: Non-ischemic cardiomyopathy
NTproBNP: N-terminale natriuretic peptide
NYHA: New-York-Heart-Association
ROC: Receiver operating characteristic
RV: Right ventricle/right ventricular
SD: Standard Deviation
TE: Echo time
TI: Inversion time
TR: Repetition time
