# Supplementary material for: Prognostic value of novel imaging parameters derived from standard cardiovascular magnetic resonance in high risk patients with systemic light chain amyloidosis
Source: J Cardiovasc Magn Reson. 2019 Aug 22;21:53. doi: 10.1186/s12968-019-0564-1 (PMC6704553; doi:10.1186/s12968-019-0564-1)
Supplement: Supplementary file 1 — Table S6. Late Gadolinium Enhancement (LGE) pattern analysis. Table S7. CMR data of patients with light-chain amyloidosis compared to different Late Gadolinium Enhancement (LGE) distribution groups (DOCX 31 kb) [file 12968_2019_564_MOESM1_ESM.docx]

**Table S6:** Late Gadolinium Enhancement (LGE) pattern analysis

| ***LGE Distribution*** | ***All*** | ***Transplant-free survivors*** | ***Composite endpoint (death or heart transplantation)*** | ***p-Value (between transplant free survivors and composite endpoint)*** |
| --- | --- | --- | --- | --- |
| Total number, n (%) | 70 (94.5) | 34 (48.6) | 36 (51.4) | 0.64 |
| Subendocardial LGE, n (%) | 26 (37.1) | 10 (38.5) | 16 (61.5) | 0.2 |
| Transmural LGE, n (%) | 25 (35.7) | 16 (64) | 9 (36) | 0.056 |
| Focal discrete patchy LGE, n (%) | 19 (27.1) | 8 (42.1) | 11 (57.9) | 0.52 |

**Table S7:** CMR data of patients with light-chain amyloidosis compared to different Late Gadolinium Enhancement (LGE) distribution groups

| ***CMR Data*** | Subendocardial LGE, n =26 | Transmural LGE, n =25 | Focal discrete patchy LGE, n = 19 | ***p-Value (between the LGE groups)*** |
| --- | --- | --- | --- | --- |
| *LAS (%)* | -7.7 ± 3.1 | -9.5± 3.3 | -8.4 ± 4.8 | 0.213 |
| MCF (%) | 51.6 ± 22. | 56.3 ± 20.9 | 61.3 ± 37.2 | 0.5 |
| LVEF (%) | 56 ± 12 | 58.4 ± 11.6 | 52.5 ± 12.1 | 0.28 |
| GLS (%) | -13.9 ± 4.9 | -13.3 ± 5.1 | -14.3 ± 6.7 | 0.82 |

Abbreviations: GLS global longitudinal strain, LAS long axis strain, LV EDVI LVEF left ventricular ejection fraction, MCF myocardial contraction fraction
