# Supplementary figures and images for: Prognostic value of novel imaging parameters derived from standard cardiovascular magnetic resonance in high risk patients with systemic light chain amyloidosis
Source: J Cardiovasc Magn Reson. 2019 Aug 22;21:53. doi: 10.1186/s12968-019-0564-1 (PMC6704553; doi:10.1186/s12968-019-0564-1)

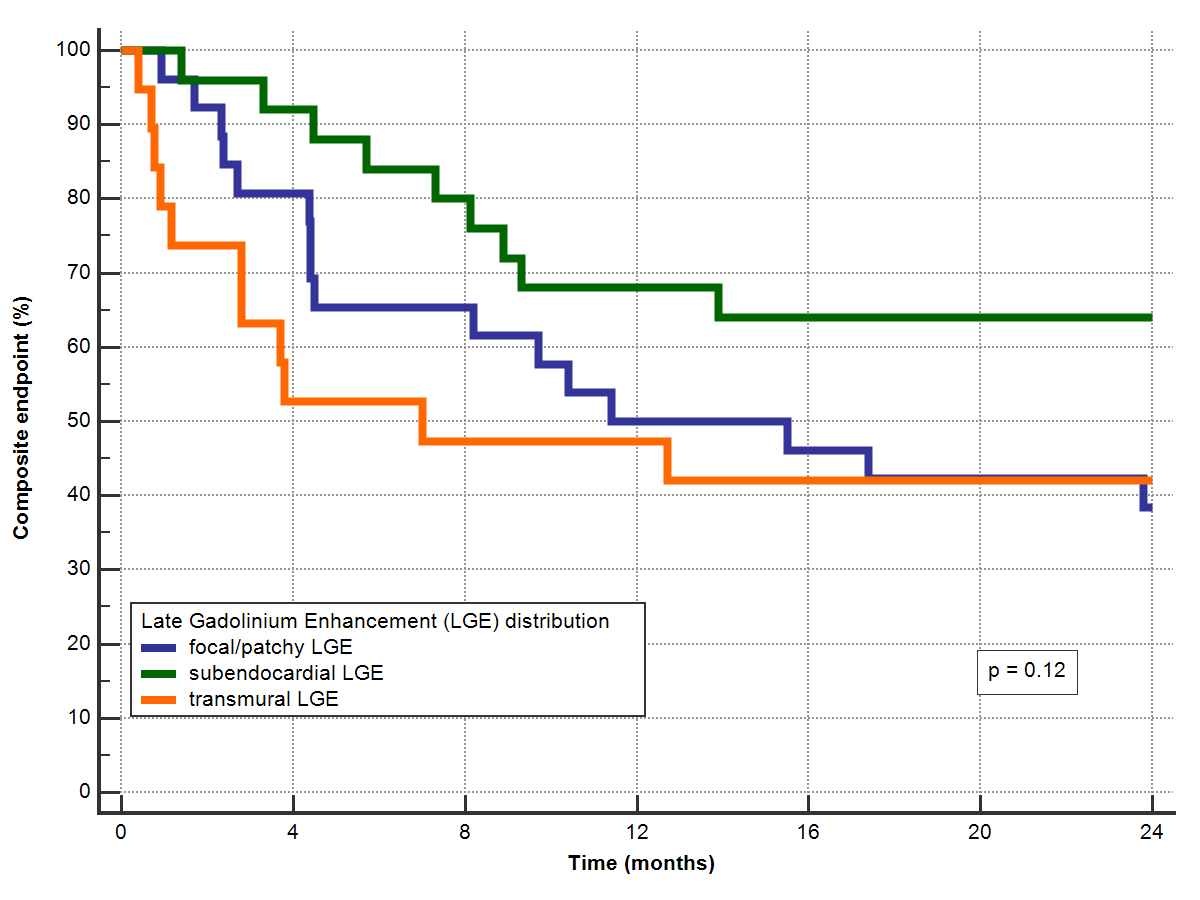

Supplement: Supplementary file 2 — Figure S6. Kaplan-Meier estimates of the time to events by late gadolinium enhancement (LGE) pattern. Presented for the composite endpoint (death and heart transplantation). (PNG 33 kb) [file 12968_2019_564_MOESM2_ESM.png]
